# Blastema from rabbit ear contains progenitor cells comparable to marrow derived mesenchymal stem cells

**Published:** 2012

**Authors:** Mohamadreza Baghaban Eslaminejad, Sima Bordbar

**Affiliations:** *Department of Stem Cells and Developmental Biology, Cell Science Research Center, Royan Institute for Stem Cell Biology and Technology, Academic Center for Education, Culture and Research (ACECR), Tehran, Iran.*

**Keywords:** Rabbit ear blastema, Mesenchymal Stem Cells, Differentiation, Proliferation, culture

## Abstract

Rabbits have the capacity to regenerate holes in their ears by forming a blastema, a tissue that is made up of a group of undifferentiated cells. The purpose of the present study was to isolate and characterize blastema progenitor cells and compare them with marrow mesenchymal stem cells (MSCs). Five New Zealand white male rabbits were used in the present study. A 2-mm hole was created in the animal ears. After 4 days, the blastema ring formed in the periphery of the hole was removed and cultivated. The cells were expanded through several subcultures and compared with the MSCs derived from the marrow of same animal in terms of *in vitro* differentiation capacity, growth kinetics and culture requirements for optimal proliferation. The primary cultures from both cells tended to be heterogeneous. Fibroblastic cells became progressively dominant with advancing passages. Similar to MSCs blastema passaged-3 cells succeeded to differentiate into bone, cartilage and adipose cell lineages. Even lineage specific genes tended to express in higher level in blastema cells compared to MSCs (*p* < 0.05). Moreover blastema cells appeared more proliferative; producing more colonies (*p* < 0.05). While blastema cells showed extensive proliferation in 15% fetal bovine serum (FBS), MSCs displayed higher expansion rate at 10% FBS. In conclusion, blastema from rabbit ear contains a population of fibroblastic cells much similar in characteristic to bone marrow mesenchymal stem cells. However, the two cells were different in the level of lineage-specific gene expression, the growth curve characteristics and the culture requirements.

## Introduction

Rabbit pinna is histologically made up of a single plate of elastic cartilage covered by a perichondrial layer and skin. It possesses an interesting capacity: potential to regenerate its injuries, a property that has increasingly lost in mammals during the evolution.^1^^,^^2^ Regeneration is a process that leads to complete replacement of the injured structures culminating in a complete restoration of tissue function, while in repair lost tissues are replaced by a scar tissue.

The regeneration process is usually investigated through experiments in which a through-and-through hole is created in rabbit ear.^3^ According to the observation made on the process following the making a hole in rabbit ear, epithelial cells migrate from the cut edge and form a sheet that is much thicker than adjacent epidermis. A few days after the injury a large blastema of fibroblastic cells extending out from the edge of cartilage appears within the epithelial sheet. It is this blastema that produces the lost cartilage in rabbit ear.^3^ According to an ultra-structural study, blastema tissue is a group of un-differentiated cells capable of dividing and differentiating into the lost tissue in wounded rabbit ear.^4^ Previous experiments have also indicated that the regeneration in rabbit ear required the proximity of the healing wound in the overlying ear skin. Skin from elsewhere on the body have been shown cannot support the regeneration.^5^ Moreover it has been shown that ear regeneration is greater in male than female rabbit and in pregnant than non-pregnant animals.^6^

Chondrocytic differentiation potential of the blastema cells in rabbit ear is obvious from the *in vivo* study conducted in this area but one important question is whether or not the cells are able to differentiate among other skeletal cell lineages such as bone or adipose cells. In other words there is no data regarding the exact nature of blastema progenitor cells. Since pinna develops from several protuberances derived from the mesenchyme of the first and second branchial arches,^7^ blastema cell would be of mesenchymal stem cell (MSC) population. Mesenchymal stem cells are adult stem cells found in varying somatic tissues. These cells are capable of differentiating into mesenchymal cells including bone, cartilage and adipose lineages. The key characteristics of MSC are their ability to form colonies that result from the activity of the cells, referred to as colony-forming unit fibroblasts (CFU-F). The frequency of CFU-F in bone marrow samples is reported to be about 1 cell per 10^4^-10^5^ mononuclear cells.^8^ Nowadays, MSCs are characterized by their ability for plastic adherence and capability of multi-lineage differentiation. In the present study, blastema progenitor cells were isolated, culture expanded and compared to MSC derived from bone marrow in terms of their differentiation potential as well as growth kinetics. Study like this would help to further understanding of the exact nature of blastema progenitor cells that have been reported to exceptionally be able to regenerate injured structures of rabbit pinna. 

## Materials & Methods

**Animals.** The use of 5 New Zealand white healthy male rabbit with 3-6 month ages was approved by the ethic committee of the Royan Institute. The animals were purchased and kept under conventional conditions in the Royan’s animal facility complex. 

**Blastema and marrow cell culture**

**Blastema. **The pinna of the rabbits was locally anesthetized using 2% lidocaine (Pasteur Institute, Iran), a circular hole with 2 mm dimensions was created in the middle of pinna and allowed forming blastema. After four days whole blastema ring was removed and incubated in Dulbecco's Modified Eagle's Medium (DMEM, Gibco, Germany) containing 10% fetal bovine serum (FBS, Gibco, Germany) and 100 IU mL^-1^ penicillin and 100 µg mL^-1^ streptomycin (both from sigma, Germany) at an atmosphere of 37 ˚C and 5% CO_2_. Migrating adherent cells from blastema explants were then lifted and propagated through several successive subcultures. Passaged-3 cells were used in our experiments.

**Marrow cells**. Under general anesthesia, 0.5 mL bone marrow were aspirated from rabbit’s tibia using 19 gauge needle, and mixed with 5 mL DMEM containing 10% FBS and 100 IU mL^-1^ penicillin and 100 IU mL^-1^ streptomycin. Bone marrow cells were washed by centrifugation for 3 minutes at 1200 rpm and followed by discarding the supernatant. The pellet was suspended in 1 mL DMEM and plated in 75 cm^2^-culture flasks at density of 10^5^ cells per mL in a 15 mL DMEM containing 15% FBS and antibiotics. The cultures were incubated in an atmosphere of 5% CO_2_ at 37 ˚C. Three days after culture initiation, the medium was changed to discard the non-adherent cells. The cultures were then allowed to achieve 70-80% connfluency. At this time they were tripsinized and subcultured at 1:3 ratio. Two additional passages were performed to obtain sufficient cells which were used to conduct the following experiments. Passage-3 cells from both blastema and bone marrow were compared in terms of their differentiation and growth characteristics.

**Differentiation assay**

**Osteogenic differentiation. **For osteogenic differentiation, confluent passaged-3 cells from either blastema cells and bone marrow tissue were added with DMEM medium supplemented with 50 µg mL^-1 ^ascorbic 2-phosphate (Sigma-Aldrich, St. Louis, MO, USA), 10 nM dexamethasone (Sigma-Aldrich, St. Louis, MO, USA) and 10 mM β-glycerol phosphate Sigma-Aldrich, St. Louis, MO, USA) for 3 weeks. At the end of this period, alizarin red staining was used to observe the matrix mineralization. For staining, the cultures were first fixed by methanol for 10 minutes and then subjected to alizarin red solution for 2 minutes. Moreover the cultures were further analyzed by Real Time PCR to compare the cells for bone-specific gene expression.

**Adipocyte differentiation**. Likewise, 70% confluent cultures of passaged-3 cells from two cultures were used to promote adipose differentiation. The differentiation-inducing medium was DMEM supplemented with 50 μg mL^-1^ ascorbic acid 3-phosphate, 100 nM dexamethasone and 50 μg mL^-1^ indomethcine. After having been in a differentiation-inducing condition for about 21 days the cells were stained with Oil red and were further analyzed by Real Time PCR for comparing two cell culture for adipose-specific gene expression. 

**Chondroblast differentiation**. For chondrogenic differe-ntiation, 2.5 × 10^5^ passaged-3 blastema and marrow cells were pelleted under 300 *g* for 5 minutes. These cells were provided with DMEM supplemented with 10 ng mL^-1^ transforming growth factor β3 (TGF- β3, Sigma-Aldrich, Deisenhofen, Germany), 10 ng mL^-1^ bone morphogenetic protein-6 (BMP6, Sigma-Aldrich, Deisenhofen, Germany), 50 mg mL^-1^ insulin transferrin selenium + premix (Sigma-Aldrich, Deisenhofen, Germany), 1.25 mg bovine serum albumin (Sigma-Aldrich, Deisenhofen, Germany) and 1% FBS. Differentiation of the culture was allowed to be extended for 21 days. At the end of this period, the pellets were processed for histological observations. For this purpose, the pellets were fixed with 10% formalin, dehydrated in ascending concentrations of ethanol, cleared in xylene, embedded in paraffin, cut into 5 μm thick sections and finally stained with toluidine blue. Some pellet was used to extract mRNA in order to further examine cartilage-specific gene expression by real time PCR.

**Quantitative real-time RT-PCR. **To quantify relative gene expression levels, total RNA was extracted from cell samples using Trizol (Invitrogen, San Diego, USA). cDNA was synthesized from total RNA using a RevertAidTM First Strand cDNA Synthesis Kit (Fermentas, Leon-Rot, Germany) according to manufacturer’s instructions. Osteocalcin, Osteopontin and ALP mRNA levels as an osteogenic differentiation marker gene, Sox9 and Aggrecan mRNA levels as a chondrogenic differentiation marker genes and Adiponectin and LPL mRNA levels as adipogenic differenti-ation marker genes were measured by real-time RT-PCR (Stepone real-time PCR Applied Biosystems, California, USA). The 20-μL reaction contained 2μL cDNA from each sample mixed with 10 μL SYBR^® ^Green PCR Mastermix (Invitrogen, San Diego, USA), 2 μL primers, and 6 μL RNase/ DNase-free water. The PCR conditions were: incubation at 95 ˚C for 2 min followed by 45 cycles at 95 ˚C for 15 sec and at 60 ˚C for 60 sec. The gene expression levels of target genes: Osteocalcin, Osteopontin, ALP, Sox9, Aggrecan, Adiponectin and LPL were determined based on the threshold PCR cycle-values (Ct (target)) following the instructions of Applied Biosystems. The relative quantification was derived using the Comparative CT Method using 2-ΔΔCt, where the amount of target normalized to an endogenous control (GAPDH) and relative to a calibrator (samples without treatment). The specific primers designed for target genes are listed in Table 1.

**Colonogenic assays. **Colonogenic assay is usually being performed to estimate the growth potential of MSC-like populations *in vitro*. In the present study, this assay was employed to determine the proliferation capacity of the isolated blastema cells and compare them with marrow mesenchymal stem cells. For this purpose, passaged-3 cells plated at 100 cells on a 10-mm Petri dish in DMEM supplemented with 10% FBS. Two kinds of cells were allowed to grow for 10 days. At the end of this period, the cultures were stained with crystal violet for about 5 minutes, observed with an inverted light microscope and the number of colonies was determined. Furthermore, the size of the colonies was determined using a microscopic objective micrometer.

**Growth curve plotting. **For this purpose, the isolated blastema and marrow cells (passaged-3) were plated in 24 well culture plate at a density of 100 cells per cm^2^ till day 13 when the culture reached confluent. During this period, the cells were counted on daily basis. Using the data, growth curve was plotted.

**Optimizing the culture condition. **In this study, initiating the cell seeding density and the FBS concent-ration of the culture medium was optimized in order to achieve extensive proliferation of the cells. For this purpose, the isolated blastema and mesenchymal stem cells (passaged-3) were plated on a 10-mm dish at varying densities of 10000، 5000، 2000، 1000، 500، and 100 cells per cm^2^ in DMEM, supplemented with 100 IU mL^-1^ penicillin, 100 µg mL^-1^ streptomycin and varying concentrations of fetal bovine serum including 5%, 10%, 15%, and 20% for a period of 10 days. At the end of this period, the cells were lifted, counted and compared. All measurements were performed in triplicate. The data was used to calculate the fold increase in the cell number of different groups. 

**Statistical analysis. **All measurements were performed in triplicate. The average was calculated and compared with one way ANOVA using SPSS version 13 (Chicago, USA). A *p* value of < 0.05 was considered as statistically significant.

**Table 1:** Primers used in the PCR

**Target Gene**		**Primers**	**Annealing Temperature**
Osteogenic marker genes	Osteocalcin	Forward primer : 5' CTCAGCCTTCGTGTCCAA 3' Reverse primer : 5' CTCGCACACCTCCCTCTTG 3'	57 ˚C
	Osteopontin	Forward primer : 5' GGCTAAACCCTGACCCATCT 3' Reverse primer : 5' GTGGTCATCGTCCTCATCCT 3'	59 ˚C
	ALP	Forward primer : 5' ACTTTGTCTGGAACCGCACT 3' Reverse primer : 5' GTGGTCAATCCTGCCTCCT 3'	58 ˚C
Chondrogenic marker genes	Sox9	Forward primer : 5' AAGATGACCGACGAGCAG 3' Reverse primer : 5' GGCTTGTTCTTGCTGGAG 3'	56 ˚C
	Aggrecan	Forward primer : 5' GGAGGTCGTGGTGAAAGGTG 3' Reverse primer : 5' CTCACCCTCCATCTCCTCTG 3'	60 ˚C
Adipogenic marker genes	Adiponectin	Forward primer : 5' CGGTGAGAAGGGTGAAAAAG 3' Reverse primer : 5' GCTGAGCGGTAGACATAG 3'	57 ˚C
	LPL	Forward primer : 5' TTCAACCACAGCAGCAAGAC 3' Reverse primer : 5' TAACAGCCAGTCCACCACAA 3'	57 ˚C
HKG	GAPDH	Forward primer : 5' GGAGAAACCTGCCAAGTATG 3' Reverse primer : 5' TGAGTGTCGCTGTTGAAGTC 3'	60 ˚C

## Results

**Fig. 1:**
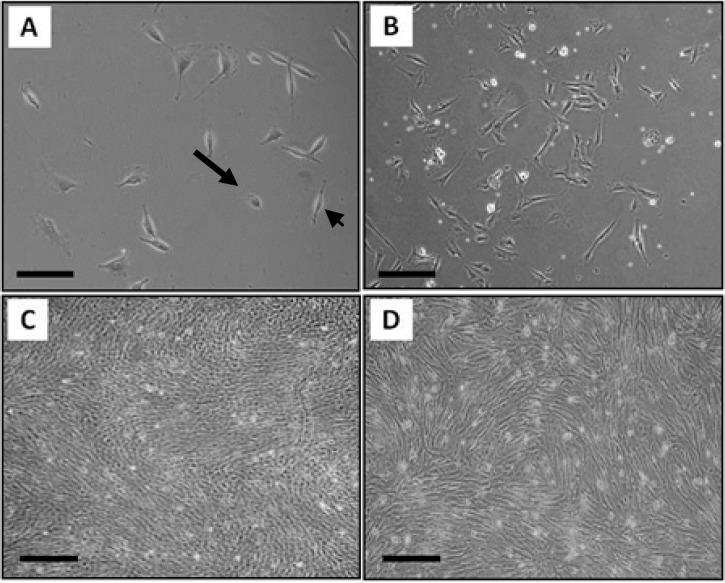
The culture of blastema and marrow cells. The primary culture of blastema (A) includes fibroblastic cells (the arrowhead) and flattened epithelial cells (the arrow) and the primary culture of marrow (B) was heterogeneous containing variety of cell morphology. Both cultures become relatively homogenous after several subcultures having fibroblastic cells (C) blastema (D) marrow; Bar= 100 µm.

**Cell culture. **Each cell type, were observed daily with phase contrast microscopy. Based on these observations about 9-12 days after culture blastema cells in primary culture start to migrate from blastema ring. The cultures include fibroblastic cells (that forms in colonies) and flattened epithelial cells (Fig. 1A). To eliminate cells that have no stem cell-like properties we passaged the culture. Progressively fibroblastic cells dominated the culture (Fig. 1C). Similarly the primary marrow cell culture appeared to be heterogeneous having variety of cell types including hematopoietic as well as fibroblastic cells (Fig. 1B). Fibroblastic cell population dominated the culture after several consecutive passages (Fig. 1D). 

**Multilineage differentiation**

**Fig. 2:**
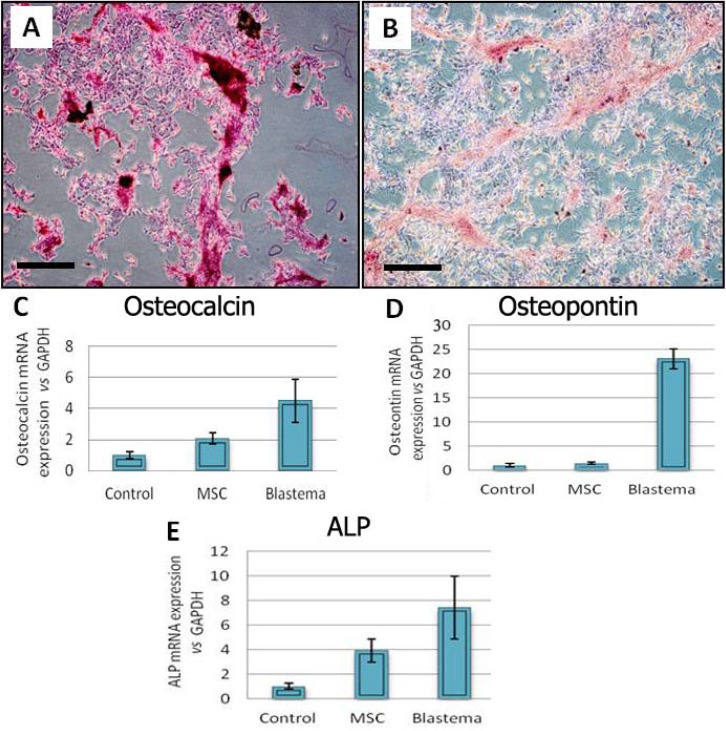
Osteogenic culture of the blastema and marrow-derived cells. (A) Alizarin red staining of the blastema cell culture. (B) Alizarin red staining of the marrow cell cultures. Blastema cells expressed ostocalcin (C), osteopontin (D) and ALP (E) in significantly higher level than marrow cells; *p* < 0.05; Bar = 100 µm.

**Osteogenic differentiation**. The observations made on the osteogenic cultures revealed the formation of multiple nodule-like structures in this cultures. First, sign of osteogenesis was observed in morphology of the cells after one week and these changes continued until 21 day. It seems that blastema cells have more and soon changes in morphology and nodule formation than MSC cells. These nodules tended to be heavily stained with alizarin red which stains mineralized matrix deposited by the cells (Figs. 2A-B). Real Time-PCR analysis of bone related genes such as osteocalcin and osteopointin and ALP (alkaline phosphatase) also demonstrate that osteogenic gene expression in blastema cells is more vigorously than MSCs cells (*p* < 0.05) (Figs. 2C-E).

**Fig. 3:**
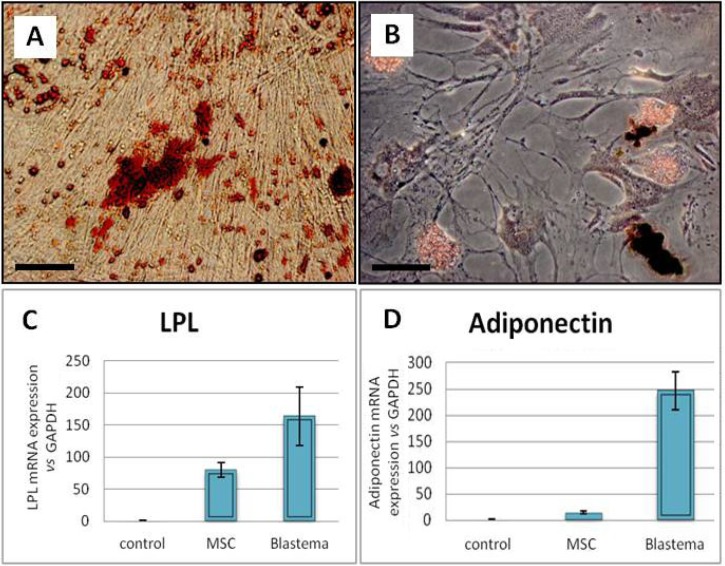
Adipogenic culture of the blastema and marrow-derived cells. (A) Oil red staining of the blastema cell. (B) Oil red staining of the marrow cell cultures. Blastema cells expressed LPL(C), and adiponectin (D) in significantly higher level than the marrow cells; *p* < 0.05; Bar = 100 µm.

**Adipogenic differentiation**. The adipogenic differentiation potential of the studied cells revealed lipid droplet-containing cells in the adipogenic cultures. These lipid droplets were stained red following the Oil red staining (Figs. 3A-B). First sign of adipogenic differentiation was observed in blastema cells after one week while these changes were obvious in MSC culture about 10 days after induction initiation. Real Time-PCR analysis of adipose related genes such as LPL(lipoprotein lipase) and adiponectin revealed significantly more expression level of the genes in blastema cells than MSC culture (*p* < 0.05) (Figs. 3C-D).

**Fig. 4:**
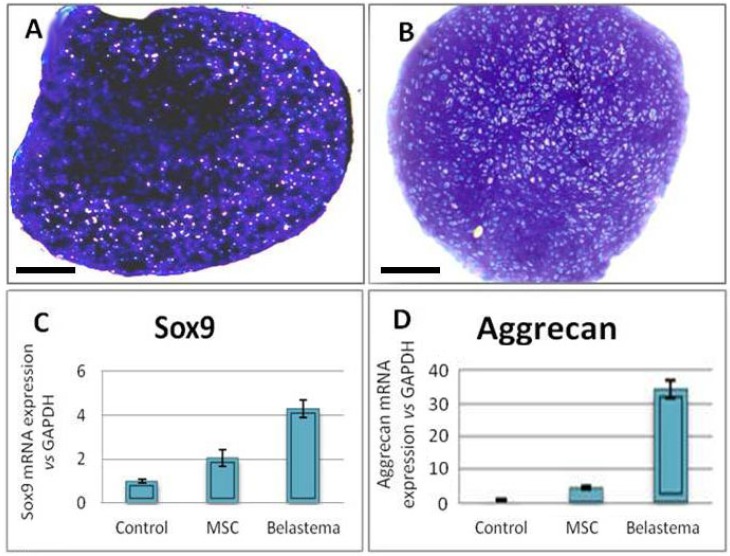
Chondrogenic culture of the blastema and marrow-derived cells. (A) Toluidine blue staining of the blastema cell. (B) Toluidine blue staining of the marrow cell cultures. Blastema cells expressed Sox9 (C), and aggreacan (D) in a significantly higher level than the marrow cells; *p* < 0.05; Bar = 100 µm.

**Chondrogenic differentiation**: Toluidine blue staining of the sections prepared from chondrogenic pellets demonstrated the presence of a metachromatic matrix at both cell cultures (*p* < 0.05) (Figs. 4A-B). Moreover Real Time-PCR analysis of chondrogenic genes such as aggrecan and sox9 demonstrated that the genes in blastema cells expressed significantly more than those in MSC culture (*p* < 0.05) (Figs. 4C-D).

**Fig. 5:**
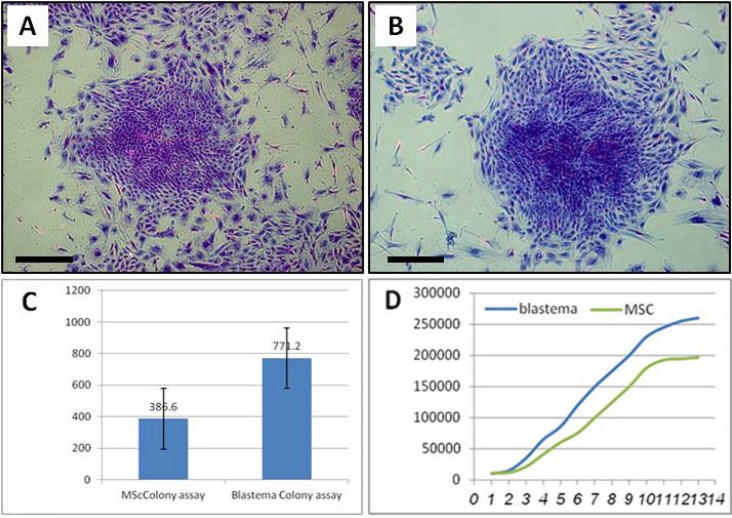
Colonogenic assay and growth curve of blastema and marrow cells. Cristal violet staining of the blastema (A) and marrow colonies (B). Blastema cells produced significantly more colonies compared to the marrow cells; *p* < 0.05 (C). Blastema cells tended to have a short lag phase than the marrow cells. They also had an extended exponential phase compared to that of the marrow cells (D); Bar = 100 µm.

**Colonogenic assay. **According to the results, 77.20 % ± 8.55 of the blastema cells tended to form colonies compared to about 38.60% ± 5.03 of MSCs. The colonies displayed several to a few hundred fibroblastic cells (Figs. 5A-C). The average size of the colonies was according to our measurement, 2.51 ± 1.04 mm^2^ in blastema cells and 1.71 ± 1.51 mm^2^ in MCS cells. Difference between two groups was statistically significant (*p* < 0.05).

**Growth curve. **According to the curve blastema cells possessed a short lag phase of about 1 day and immedi-ately entered into exponential growth phase. They reached into plateau at day 12. Mesenchymal stem cells exhibited a lag of 2 days after which they entered into proliferation. Their plateau was reached at day 10. Therefore, MSC had a short period of proliferation phase (Fig. 5D). 

**Fig. 6:**
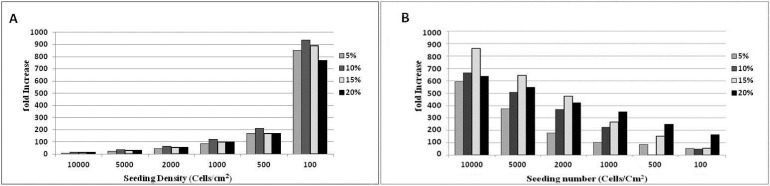
Culture requirement of the blastema and marrow cells for extensive proliferation. A) While blastema cells undergo extensive proliferation in presence of 10% FBS at 100 cell per cm^2^ density (*p *< 0.05)(A) those of marrow present optimal proliferation in a medium containing 15% FBS with 10000 cell per cm^2^ density (*p *< 0.05) (B).

**Optimizing the culture condition. **According to the data, significantly more fold increase occurred in the culture initiated at 100 cell per cm^2^ and 10% FBS in blastema cells, and 10000 cell per cm^2^ and 15% FBS in MSCs (*p* < 0.05) (Figs. 6A-B).

## Discussion

In the present study blastema cells involved in healing of rabbit ear wound isolated, culture expanded and characterized in comparison to mesenchymal stem cell derived from the same rabbit marrow tissue. Our findings indicated the blastema cells from rabbit ear were comparable to marrow MSCs in terms of both growth characteristics as well as multi-lineage differentiation potential into three skeletal cell lineages. According to our data blastema cells even appeared to be more colonogenic than marrow MSCs. They also expressed lineage specific genes in more level than marrow MSCs did. These data indicate that the repair cells in rabbit pinna are from MSC population. In the former studies blastema is described to be made up of a set of undifferentiated cells capable of producing the cartilage component of rabbit ear.^3^^-^^6^ The exact nature of the cells has remained to be identified.

MSCs from marrow as well as other sources including adipose tissue, cord blood, amniotic fluid, peripheral blood, bone, cartilage, and muscle tissues were described as colonogenic cells with fibroblastic morphology.^9^^-^^15^According to our observation the blastema cell culture first contained varying cell morphology but with advancing passages those cells that were not able to withstand culture condition as well as those with limited proliferation capacity were eliminated. The fibroblastic blastema cells were the only one that resist the culture conditions and tended to survive and dominate the culture. Some study has indicated that stem cells are much resistant to culture stressful conditions.^16^ The fibroblastic cells dominated the blastema culture were highly colonogenic. These findings all indicated the stem cell nature of the isolated cells in this study.

In contrast to hematopoietic stem cells that possess a single reliable marker MSCs lacks a specific single markers. These cause the isolation as well as identification of the MSCs to become more difficult task. To solve this problem tissue specific and mesenchymal stem cell committee of international society for cell therapy has proposed the minimal criteria to identify MSCs from human tissue. These include: 1- The cells must be plastic adherence; 2- They must possess a tripotent differentiation potential into bone, cartilage and adipose cell lineages; and 3-The must express CD73, CD105 and CD90 and not express hematopoietic and endothelial markers. For animal studies the presence of the first two criteria is sufficient.^17^ In the present study both cells (blastema and marrow MSCs) were evaluated in terms of tripotent differentiation potential. Furthermore according to our observations both cells tended to be plastic adherent. Therefore either cell was of MSC population described elsewhere.

According to the differentiation experiments blastema cell tended to express osteogenic, adipogenic and chondrogenic-specific genes in higher level than marrow MSCs. These mean that they possess more differentiation potential than MSCs. *In vitro* differentiation of MSCs is of utmost importance regarding their application in regenerative medicine. One strategy in cell-based treatment of tissue defect believes that the cells intended to be transplanted must be fully differentiated in order to avoid their unwanted differentiation in the defect site.^18^ More expression of differentiation-specific genes can be resulted from two possible ways: 1- An extensive expression of the genes in each single cell; 2- More cell involvement in the expression process. In the present work the cell number differentiated at culture was not quantified. Therefore the mechanism of the higher expression of genes in blastema-derived cells is not clear and needs further investigations.

Marrow MSCs and blastema cells were according to our finding different with respect to their *in vitro* requirement for extensive proliferation. While marrow cells achieved higher proliferation in medium containing 15% FBS those of blastema reached maximum proliferation in medium with 10% FBS. Moreover marrow cells produced a fewer colonies in culture than the blastema cells did. Growth curve study results were also indicative of some difference between the two cell types. Considering these data blastema cell are not identical but rather similar to marrow MSCs. 

In conclusion, blastema developed in periphery of the holes punched in rabbit ear contains a cell population that is comparable to mesenchymal stem cells derived from bone marrow tissue of the same rabbit. Blastema-derived cells express significantly more lineage specific genes than marrow MSCs in adipogenic, osteogenic and chondrogenic cultures. They also propagate in rapid rate than MSC in culture.
